# Effect of Polymers on the Physicochemical Properties and Biological Performance of Fenoprofen Calcium Dihydrate-Triacetyl-β-Cyclodextrin Complex

**DOI:** 10.3390/pharmaceutics9030023

**Published:** 2017-07-03

**Authors:** Hussein O. Ammar, Tarek S. Makram, Shaimaa Mosallam

**Affiliations:** 1 Pharmaceutics and Pharmaceutical Technology Department, Future University, Cairo 11311, Egypt; husseinammar@fue.edu.eg; 2 Pharmaceutics Department, October 6 University, Giza 12511, Egypt; shaimaamosallam@o6u.edu.eg

**Keywords:** fenoprofen calcium dehydrate, triacetyl-β-cyclodextrin, hydroxypropylmethyl cellulose, ethyl cellulose, sustained release

## Abstract

*Background*: Fenoprofen calcium dehydrate (FCD) is counted as a non-steroidal, anti-inflammatory, anti-arthritic drug. FCD is slightly water soluble. It is indicated for mild pain relief, where the suggested dosage is 200 mg orally every 4 to 6 h. *Aim*: Reduce dissolution efficiency, reach an extended therapeutic effect and reduce the frequency of the drug side effects. *Method*: Combination of the co-evaporated drug:triacetyl-β-cyclodextrin complex prepared in a ratio of 1:3 and either of two polymers—hydroxylpropylmethyl cellulose (HPMC) or ethyl cellulose (EC)—in the same formulation. In vitro dissolution studies were carried in simulated gastric (pH 1.2) and intestinal (pH 6.8) fluids, by using the USP dissolution tester (rotating paddle apparatus). The FCD in vitro release from EC/drug complex was markedly retarded. Interaction between fenoprofen, TA-β-CD, EC, HPMC in the solid state were confirmed by FT-IR, DSC, XRD and SEM. In vivo studies assessed the anti-inflammatory and analgesic activities and the results were compared with the market product Nalfosab^®^ Capsules. *Results*: Remarkable inhibition of inflammation and nociception after 24 h was attained for EC/drug complex. *Conclusions*: EC/drug complex has a sustained effect due to high remaining amount after elapsing with remarkable inhibition of inflammation.

## 1. Introduction

Fenoprofen calcium dihydrate (FCD) is counted as a nonsteroidal, anti-inflammatory, anti-arthritic drug. FCD is slightly soluble in water, with pK_a_ 4.5 at 25 °C. FCD is highly recommended to treat mild to moderate pain at a dosage of 200 mg orally every 4 to 6 h. In case of some specific cases, such as rheumatoid arthritis or osteoarthritis, the prescribed oral dose is 300 to 600 mg, 3 or 4 times per day [1]. Fenoprofen calcium is voluntarily absorbed via the gastrointestinal tract; bioavailability being about 85%, but food and milk may reduce the time and amount of absorption. Peak plasma concentration of 50 μg/mL occurs one to two hours after a single dose [1]. The half-life in plasma is about 3 h. Fenoprofen calcium is about 99% bound to plasma proteins. Excretion of FCD in urine reaches 90% of the dose in 24 h [2].

Cellulose ether (CE) is a cellulose derivative considered an abundant polymer in Nature. It is also a water-soluble polymer. Its products are used as binders, thickeners, water-retention agents and film formers. They also function as suspending agent, protective colloids, lubricants, emulsifiers, and surfactants [3].

Cellulose ether derivatives exist in a number of forms, but there are mainly two essential types: hydroxypropylmethyl cellulose (HPMC) and ethyl cellulose (EC). HPMC as a hydrophilic gel matrix and bio-adhesion material that is widely used in pharmaceutical preparations. That’s why it is selected by most formulators probably due to the claim that it gives fast gel formation to control initial drug release and also controls further release due to its strong viscous gel [4]. Its popularity can be attributed to its non-toxic nature, ease of compression, and capability to accommodate a high level of drug loading [5,6]. Based on the difference of molecular weight and viscosity, it is equipped with characteristics and application of emulsification, bonding, thickening and adhesion, suspension, gelation and film forming. HPMC is used in pharmaceutical products, for example as tablet coating, in granulation and controlled release [3] Ethyl cellulose (EC) has been used in pharmaceutical formulations for various purposes, such as taste-masking of bitter actives , moisture protection, stabilizer, extended release multiparticulate coating, micro-encapsulation of actives, extended release binder in inert matrix systems, solvent and extrusion granulation . The application of EC in wet extrusion processes is limited, since the polymer has considerable elastic properties, but can be successfully used as matrix former combination with some suitable plasticizing agents [6,7]. EC is an ideal polymer for the formation of products allowing modified drug release. It is insoluble at any pH that occurs in organism, but in the presence of the gastric juice it undergoes swelling. It then becomes permeable for water and permits extended modified drug release. This makes it suitable for ensuring improved patient compliance. A small number of EC polymers have been approved for general pharmaceutical application and are used in extended release solid dosage formulations. Several types of such EC exist, e.g. Ethocel 4, Ethocel 10 and Ethocel 45, which differ in the length of the polymer chains, the rate of dissolution, and the viscosity of their solution. EC is suitable to prepare maintenance release (MR) coatings [8,9,10,11].

Hydrophobic cyclodextrin is considered a suitable slow dissolving carrier, i.e. triacetyl-β-cyclodextrin (TA-β-CD) [12] can be used in the demonstration of FCD dissolution profiles. CDs can also be used along with other carrier materials to optimize drug release rates. Improved nifedipine bioavailability with reduced first pass metabolism was observed with a modified oral dosage form containing a fast release portion of the drug with HP–β-CD and HCO-60, a nonionic surfactant (i.e., an amorphous drug form obtained by spray drying with the CD and surfactant) and a slow release portion with hydroxypropyl celluloses (HPCs) of different viscosity grades [13]. Combinations of drug complexes with hydrophilic and hydrophobic CDs in appropriate ratios can be a promising drug delivery system for prolonged therapeutic effect and balanced bioavailability. In rabbits, a sustained release nicardipine formulation, developed by mixing the drug complexes with HP-β-CD (fast release fraction) and with hydrophobic TA-β-CD (sustained releasing portion) in appropriate ratios, showed markedly retarded drug release with prolonged maintenance of plasma levels [14]. A sustained release 2-layered nifedipine tablet formulation was developed by using the drug complexes with β-CDs and HP-β-CDs [15]. Use of CDs with a hydroxyapatite matrix was indicated to control the release of chemotherapeutic agents containing toxic metals, such as rhodium (II) citrate and butyrate, and to provide localized antitumor chemotherapy with minimal side effects [16].

Consequently, in this paper it was important to study a combination of FCD and hydrophobic carrier. The second step is to provide a suitable modulation of FCD sustained release formula. HPMC and EC were chosen to achieve the set goal. Characterization studies, like DSC, FTIS, XRD and SEM, of drug complex, MC/drug complex and EC/drug complex have been done. The release behavior of the three drug formulations in vitro and in vivo were examined, predicting the usage of these formulas as a prolonged release drug carrier.

## 2. Materials and Methods

### 2.1. Materials

FCD (M_W_ = 558.65) was purchased from Lanxi Long Term company (Lanxi, China). Hydroxypropylmethyl cellulose (90 HG) and ethyl cellulose (ethoxyl content 48%, 10 CPS) were bought from Sigma-Aldrich (St. Louis, MO, USA) and Acros Organics Company (Geel, Belgium), respectively. Trisodium phosphate 12-hydrate (M_W_ = 380.12) was kindly supplied by Carlo ErBA (Milano, Italy). The rest of the chemicals and solvents used were of reagent grade.

### 2.2. Methods

#### 2.2.1. Preparation of Polymer-Drug Complex Blend

The optimum drug complex which exhibits the lowest release and dissolution efficiency was identified (Fenoprofen calcium dihydrate: TA-β-CD in 1:3 ratio (Mwt/Mwt*)* prepared via a co-evaporation method) as stated in a previous study [12]. For better results, it was further separately mixed with two different polymers, HPMC and EC, in a ratio of 4:1 drug complex to polymer (*w/w*) by the same method [17]. The prepared drug complex was dissolved in an adequate amount of ethanol, while HPMC and EC were dissolved in the least amount of water and ethanol, respectively. The HPMC or EC solution was added to the FCD: TA-β-CD complex solution with continuous stirring. At room temperature, the produced mixture was stirred for 24 h. Then, it was evaporated to dryness under vacuum. Consequently, the end product was sieved by a 63–160 μm granulometric sieve. The method of preparation followed previous reports [17,18,19], with some modifications.

#### 2.2.2. In Vitro Dissolution Studies

Dissolution studies were done in triplicate using a USP dissolution tester (rotating paddle apparatus) at 100 rpm. That was performed at 37 ± 0.5 °C in 750 mL of 0.1 N HCl (pH 1.2). The duration was two hrs, and after that, in phosphate buffer (pH = 6.8) for a period of six hrs after changing the pH from 1.2 to 6.8 via addition of 250 ml of 0.20 M tribasic sodium phosphate [20]. Fenoprofen calcium dihydrate and its equivalent in HPMC/drug complex and EC/drug complex were filled into size 00 capsules. Each capsule was positioned at the base of the dissolution cell to avoid floating [21,22,23,24]. Five milliliter samples were taken after 1, 2, 3, 4, 5, 6, 7 and 8 h. The samples were passed through a 0.22 μm filter. Immediately, the volume was preserved in the vessel by means of new dissolution medium [25]. The samples analysed for FCD content were measured for the absorbance at predetermined *λ*_max_ 270.8 nm against 0.1N HCl as blank for the first 2 h and at *λ*_max_ 270.6 nm against phosphate buffer (pH = 6.8) as a blank designed for the remaining 6 h. The dissolution drug profiles were characterized using the Dissolution Efficiency (DE) parameter. DE is determined by the calculating the area below the dissolution curve for a specific period of time using trapezoidal method, expressed as a percentage of the area of the rectangle that represents dissolution of 100% at the identical time interval [26,27,28].

#### 2.2.3. Characterization of Drug Complex and Polymer-Drug Complex Blends

Several techniques were used to distinguish the prepared complex of the drug with TA-β-CD and polymer-drug complex blends. These techniques were also used to draw the changes in drug physicochemical properties; chemical interactions and crystallinity. These techniques are Fourier-Transform Infrared Spectroscopy (FT-IR), Differential Scanning Calorimetry (DSC), Scanning Electron Microscopy (SEM) and X-Ray Diffractometry (XRD).

##### Differential Scanning Calorimetry (DSC)

Thermal properties of the samples were monitored and evaluated via DSC-50 (Shimadzu, Kyoto, Japan). The procedure was done by putting the samples in sealed aluminum pans and then heated during a flow of nitrogen of a rate of 10 mL/min. Almost 1 mg of the examined samples was evaluated at an increasing temperature rate of 10 °C/min [28,29] starting from 25 °C up to 320 °C. The three formulae thermograms and the plain drug samples were compared. Temperature and heat flow calibration was achieved with indium.

##### Fourier-Transform Infrared Spectroscopy (FT-IR)

It was essential to monitor any possible interactions between the drug and the carrier via Infrared (IR) spectroscopic analysis. KBr disks were used to record the infrared spectra on a FT-IR spectrometer (IR affinity-1, Shimadzu, Kyoto, Japan). The procedure was summarized in the four following steps: first, grinding finely the sample with KBr, secondly, insertion into the sampling cup, then, smoothing the powder and finally, compressing it using a compression gauge. The obtained sample was positioned in the light path and the spectrum was gained. Individual TACβD, FCD, HPMC and EC were run as controls. The range of scanning was reserved from 4000 to 400 cm^−1^ at a resolution of 4 cm^−1^ [30].

##### X-ray Diffractometry (XRD)

The consequences of the inclusion complex phenomena were examined by XRD (Diano X-ray diffractometer, Wobum, MA 01801, USA) on crystallinity of drug-powdered samples. That was done under specific conditions which are Ni-filtered (Cu-Kα) radiation, at 45 KV voltages and 40 mA current. The rate of scanning used was 2° min^−1^ over the 4° to 50° diffraction angle (2θ) range [29].

##### Scanning Electron Microscopy (SEM)

The samples morphological features were investigated by means of SEM (JXA-840A Jeol, Tokyo city, Japan). The powder was earlier preset on a brass stub with double-sided adhesive tape. After that, it was coated with a thin gold layer under vacuum conditions to be electrically conductive (300 °A), at 30 W for a duration of 30 s. The obtained pictures were at a magnification of 1500× and an excitation voltage of 15 KV [18].

### 2.3. In Vivo Studies

#### 2.3.1. Assessment of Anti-Inflamamtory Activity (Carrageenan-Induced Edema)

Rat hind paw edema was induced by carrageenan. It was used to evaluate the acute anti-inflammatory activity of the three formulae by injection of an irritant (phlogistic agent) into the tissues of the plantar surface of the hind paw of the rat [31]. The experiment was done using one dose level of the standard and test groups. Rats weighing 150–200 gm, were fasted for 16 h before starting the experiment and divided into vehicle and control, market product and test groups of six animals each. The inflammation was induced in rat paws by injection of 0.1 mL of 1% carrageenan suspension in 0.9% NaCl solution into the sub-plantar tissue of the hind paw. At the beginning of the experiment, the paws thickness (baseline) of all animals was measured using a Vernier caliper. The first group was kept as a vehicle control group, injected of the sub-plantar tissue of the right hind paw by 0.1 mL of 0.9% NaCl solution. While the second group injected by 0.1 mL of 1% carrageenan suspension in 0.9% NaCl solution in the same location and served as carrageenan positive group. The standard group received a commercial fenoprofen calcium dihydrate product (Nalfosab^®^ Capsules, Sabaa Company, Alexandria, Egypt) in a dose equivalent to 45 mg/kg fenoprofen calcium dihydrate orally [32,33,34,35,36]. In the test groups, the three formulae were administrated orally using the same dose of fenoprofen calcium dihydrate given to the standard group [37]. Following the drug administration by an hour, the inflammation was induced by repeating the previous inflammatory inducer. Meanwhile, the left paw was kept as a reference [38].

The anti-inflammatory effect of the examined preparations were estimated by monitoring and evaluating the magnitude of paw swelling in the pretreated animals with those induced in control animals receiving saline, and carrageenan in standard groups. The measurement was carried out at 2, 6, 12 and 24 h after administration of the medicated formulae. The percent change in paw swelling (% edema) compared to baseline measurement was taken as the criterion of comparison [31]. The inhibition % of the induced edema was used as an indicator to measure the anti-inflammatory effect in comparison with the control [39]: % edema = (test paw thickness − initial paw thickness)/(initial paw thickness) × 100 [40] and % inhibition of inflammation = [(control % edema − test % edema) × 100]/(control % edema) [40,41].

#### 2.3.2. Assessment of the Analgesic Activity (Writhing Test)

In this test, groups of mice, each comprised of six male albino mice weighing 25 ± 5 g were used. One group received no treatment (control) and the others received the three medicated formulae or the commercial Nalfosab^®^ Capsules orally in a dose equivalent to 30 mg/kg fenoprofen calcium dihydrate [37]. Acetic acid was used to induce abdominal constriction upon which the analgesic effect of the samples were monitored and evaluated [42]. The test was performed with some modification as described by Adzu et al. [43]. Ten mL/kg of 0.6% acetic acid solution was injected into the mice through the intraperitoneal route (ip) 2, 6, 12, 24 h after administration of the medicated formulae. To facilitate the observation process the mice were located individually in transparent observation cages. Acetic acid induced abdominal constriction caused stretching of hind limbs that took place between 5 and 15 min and was cumulatively counted. The analgesic effect was articulated as a percent of inhibition of nociception (reduction in episodes of writhing) comparing control and treated groups [44,45]: % inhibition of nociception = [(number of writhes of control − number of writhes of test) × 100]/(number of writhes of control).

## 3. Results and Discussion

### 3.1. Effect of Polymers on the Dissolution of Fenoprofen Calcium Dihydrate

**Figure 1:**
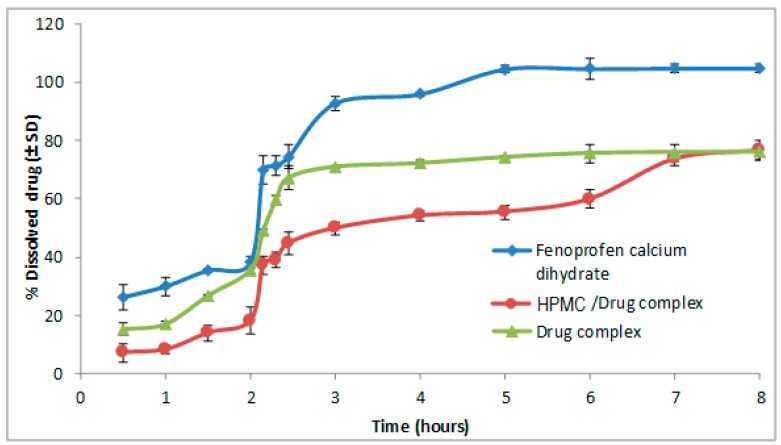
Effect of HPMC/drug complex on the dissolution of fenoprofen calcium dihydrate at pH = 1.2 for 2 h and then continued in pH = 6.8 for 6 h.

**Figure 2:**
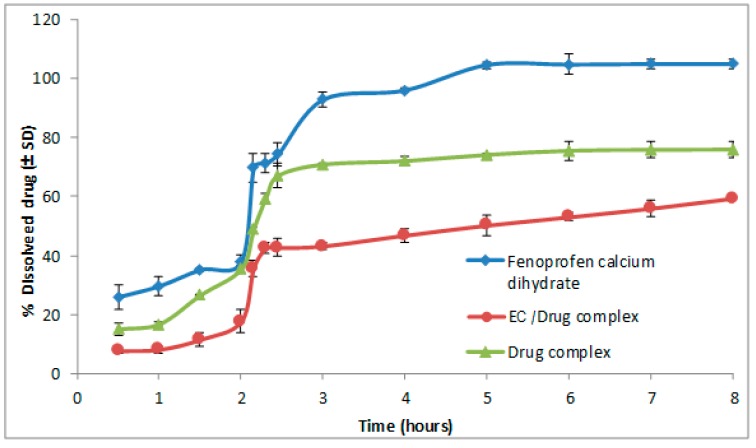
Effect of EC/drug complex on the dissolution of fenoprofen calcium dihydrate at pH = 1.2 for 2 h and then continued in pH = 6.8 for 6 h.

The presence of hydrophilic swelling polymer (HPMC), as a portion of HPMC polymer, resulted in a limited slowing down of the drug release rate to reach 77% after 8 h (Figure 1). On the other hand, the EC water insoluble polymer increased the reduction of drug release rate, to reach less than 60% after 8 h (Figure 2). The use of individual polymers allowed an improved modulation of drug release rate with value to TA-β-CD formulations alone. It was observed upon comparing the effect of drug release rate between the EC and the HPMC polymer that EC polymer had a more significant reducing effect than that with HPMC.

Dissolution efficiency was used for comparison between the complex mixed with HPMC and the corresponding one with EC. The lowest dissolution efficiency value was attained for the complex mixture with EC.

### 3.2. Characterization of Drug Complex and Polymers/Drug Complex

#### 3.2.1. Differential Scanning Calorimetry (DSC)

**Figure 3:**
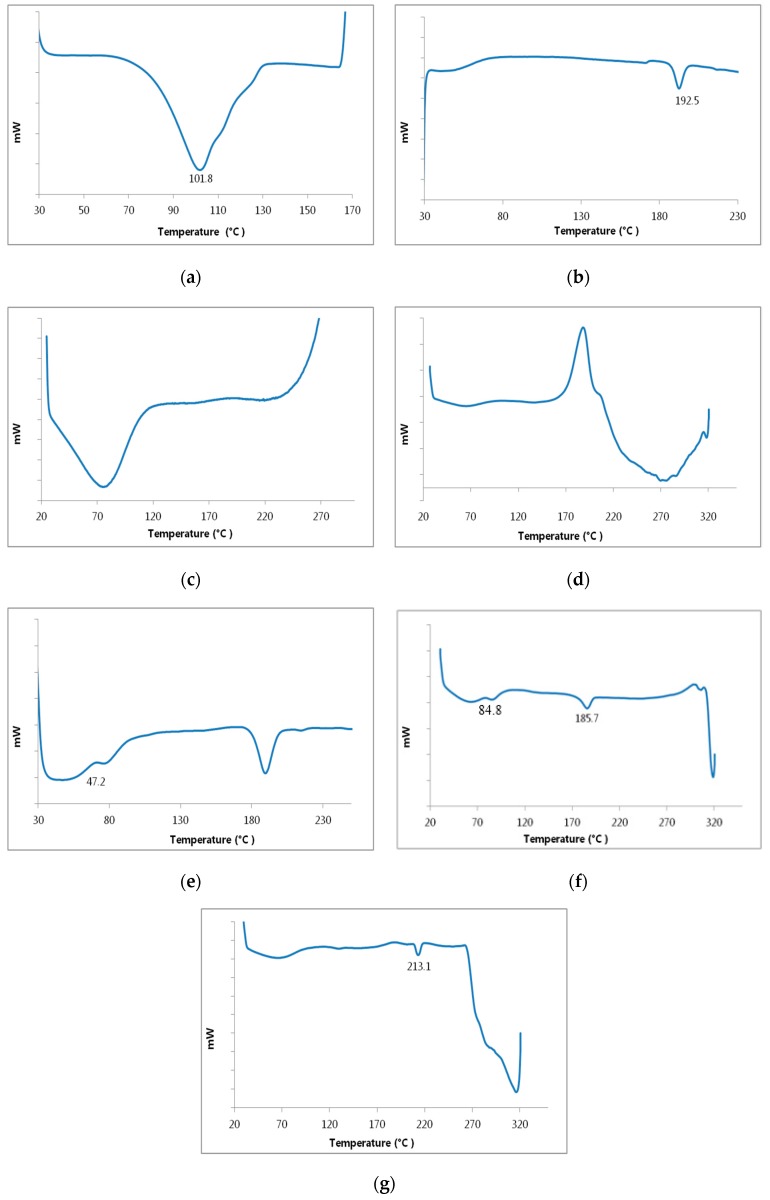
Differential scanning thermograms of: fenoprofen calcium dihydrate (**a**); TA-β-CD (**b**); HPMC (**c**); EC (**d**); drug- TA-β-CD complex 1:3 (**e**); HPMC/drug complex (**f**); EC/drug complex (**g**).

The thermal alteration property of the drug complex and polymers/drug complex were examined and detected via DSC with regard to the pure substances [18]. The DSC curves of fenoprofen calcium dihydrate, TA-β-CD, HPMC, EC, drug complex, HPMC/drug complex and EC/drug complex are shown in Figure 3. The observed melting point of pure FCD in the thermal curve was indicated by a sharp endothermic peak at 101.8 °C. This indicates the crystallinity of the drug and followed by the degradation of substance. On the other hand, the main factor that attributed to the loss of the weak hydrogen bonded water was the initial broad endothermic band of commercial TA-β-CD in its thermal profile.

It is worth mentioning that after which the fusion peak of a low–melting anhydrous polymorph occurred at 192.5°C. Subsequently, it recrystallizes into a form with a higher melting point, that has a fusion endothermic peaked at 219.8 °C [46].

The DSC profiles of EC and HPMC were considered as a typical form of amorphous substances, characterized by a great dehydration band within the temperature range starting from 50 up to 120 °C [17].

It is obvious that the thermogram of the co-evaporated complex prepared in a ratio 1:3 (drug: TA-β-CD) demonstrates an change and a reduced intensity endothermic peak of drug at 47.2 °C and the fusion peak corresponding to TA-β-CD was shifted to 189.6 °C. These observations may be considered a strong sign for the creation of genuine inclusion complexes [47,48].

The characteristic peak of the drug shifted to 84.8 °C and 65.2 °C for HPMC/drug complex and EC/drug complex, respectively. The fusion peak of TA-β-CD shifted to 185.7 °C in the case of HPMC/drug complex. However, the thermal curve of EC/drug complex illustrated the loss of the fusion peak of TA-β-CD with the appearance of an endothermic effect at 213.1 °C. These results confirm to the presence of polymers and drug complex interactions.

#### 3.2.2. Fourier Transform-Infrared Spectroscopy (FT-IR)

**Figure 4:**
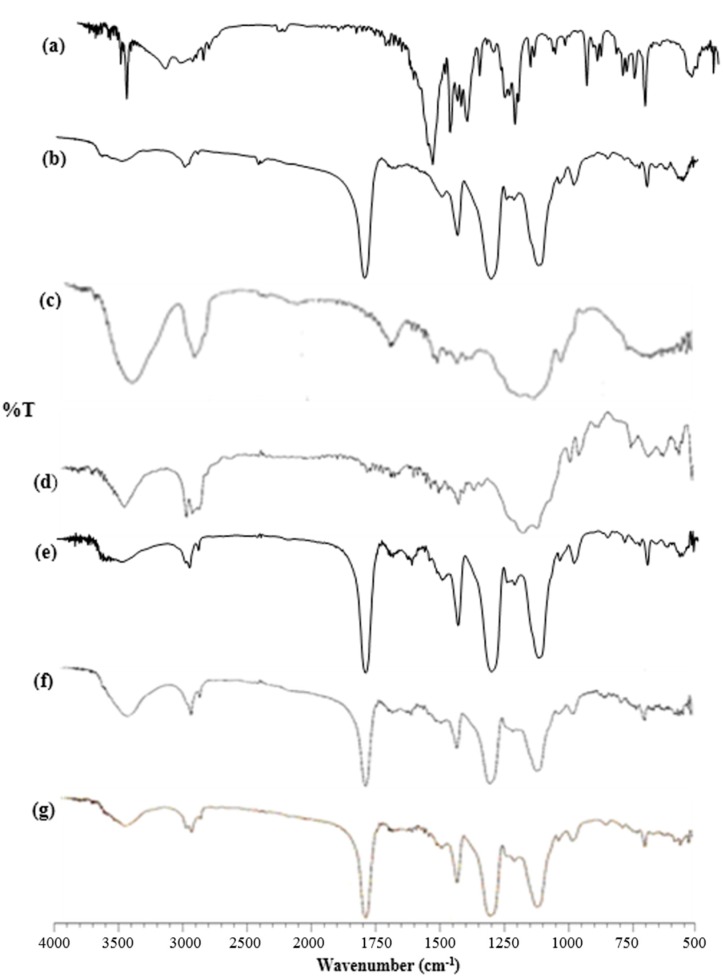
IR spectra of: fenoprofen calcium dihydrate (**a**); TA-β-CD (**b**); HPMC (**c**); EC (**d**); drug-TA-β-CD complex 1:3 (**e**); HPMC/drug complex (**f**); EC/drug complex (**g**). (ν C–O–C asymmetric ether stretching), 933–696 cm**^−^**^1^ (ν primarily aromatic out of plane bending) [49]. TA-β-CD exhibited a very strong absorption band at 1751 cm**^−^**^1^, due to C=O vibration of the acetyl (OCOCH_3_) groups [50].

FT-IR spectroscopy was used to confirm a strong interaction between drug and TA-β-CD and between drug complex and polymers. FT-IR spectra of fenoprofen calcium dihydrate, TA-β-CD, HPMC, EC, drug complex, HPMC/drug complex and EC/drug complex are shown in Figure 4.

The infrared spectrum of fenoprofen calcium exhibited the characteristic bands corresponding to the drug’s functional groups at 3649, 3601, 3284 cm**^−^**^1^ (ν –OH stretching of the hydrate), 1558 cm**^−^**^1^ (ν –O–C=O asymmetric and symmetric stretching), 1265, 1224, 1211 cm**^−^**^1^. It was observed from the FT-IR spectra of HPMC, that the peak is at 3500 to 3400 cm**^−^**^1^. That is a strong indicator of ν –OH stretching. Due to the presence of C-H bonds in the methyl and hydroxypropyl groups, an extension and contraction in phase was observed at 2900 cm**^−^**^1^ [51]. It was also observed that at 1052 cm^1^ and at range 2880−2970 cm**^−^**^1^, EC displays characteristic absorption bands for –C–O–C– stretching vibration and C-H stretching, respectively. The absorption at 1369 cm**^−^**^1^ indicates C–H bending [52,53].

In the IR spectrum of drug complexes, the characteristic ν (–OH) stretching of the hydrate at 3649, 3601, 3284 cm**^−^**^1^ and the primarily aromatic out of plane bending at 933–696 cm**^−^**^1^ were not detected, while the ν (–O–C=O) asymmetric and symmetric stretching at 1558 cm**^−^**^1^ was clearly decreased in intensity. The characteristic ν (–C–O–C) asymmetric ether stretching at 1265, 1224, 1211 cm**^−^**^1^ was not observed due to the broad TA-β-CD band at 1236 cm**^−^**^1^. It was proven that the produced complex had no chemical bonds due to the indicated results [54].

In IR spectrum of HPMC/drug complex and EC/drug complex, the characteristic bands equivalent to the functional groups of the drug were not detected. The presence of interaction between the functional group of the drug and its entrapment in the polymers and CD cavity were significantly observed.

#### 3.2.3. X-ray Diffractometry (XRD)

**Figure 5:**
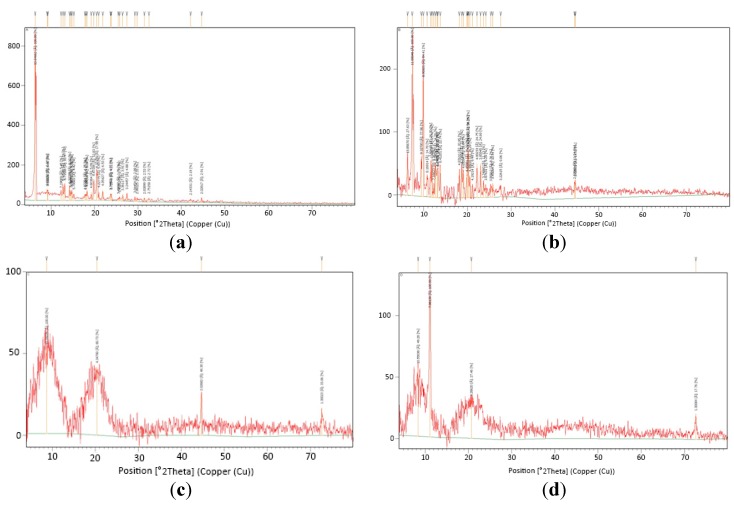

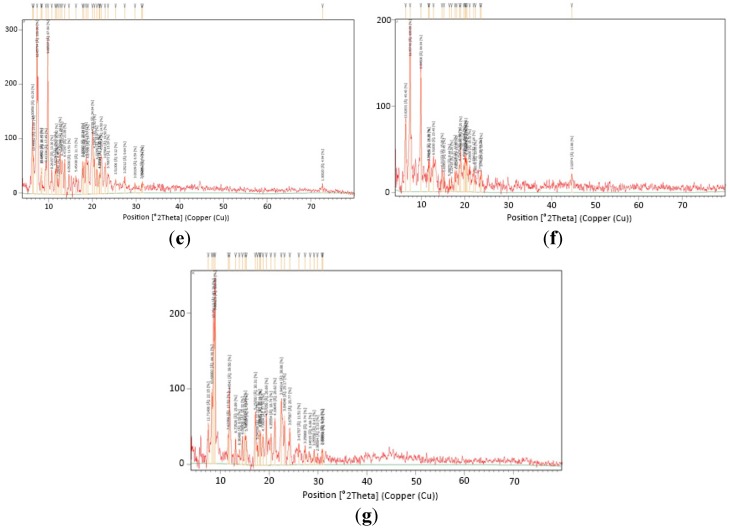
X-ray diffraction patterns of: fenoprofen calcium dihydrate (**a**); TA-β-CD (**b**); HPMC (**c**); EC (**d**); drug-TA-β-CD complex 1:3 (**e**); HPMC/drug complex (**f**); EC/drug complex (**g**). Note: The red color in each graph is characteristic for each substance respectively as shown in the figure caption and the yellow color is just a mark to indicate the peaks position and heights.

The mai+n reason of using X-ray diffraction studies was to inspect the crystallinity and supply more evidence of complex formation. The powder X-ray diffraction patterns of pure Fenoprofen Calcium Dihydrate, TA-β-CD, HPMC, EC, Drug complex, HPMC/drug complex and EC/drug complex are depicted in Figure 5.

The diffractogram for the crystalline fenoprofen calcium dihydrate showed its strongest diffraction peak as a split peak centered around 6.3° 2θ. Other peaks were observed at the diffraction angle of 2θ at 19.8° and 20.5° [55]. TA-β-CD, HPMC and EC also exhibit many characteristic peaks due to their crystalline nature.

The drug complex illustrated, in respect to the diffraction patterns of the starting materials, prominent strength diminution of some fenoprofen calcium dihydrate peaks, particularly these positioned at 6.3° (2θ). These observations may be indicative of formation of complex and suggest the inclusion process of fenoprofen calcium dihydrate in TA-β-CD cavity [56,57]. In contrast to the above observations, HPMC/drug complex showed marked decrease in the characteristic peak of the drug. These results showed the existence of a new solid phase with lower degree of crystallinity, that could be created by the molecular interaction of the HPMC and the drug complex [58,59]. The EC/drug complex offered a diffraction pattern totally diffused, with the loss of the characteristic peak of FCD.

The previous results proved that the formation of a new solid phase which indicates strongly an inclusion between EC and drug complex in the solid state [59,60,61].

#### 3.2.4. Scanning Electron Microscopy (SEM)

**Figure 6:**
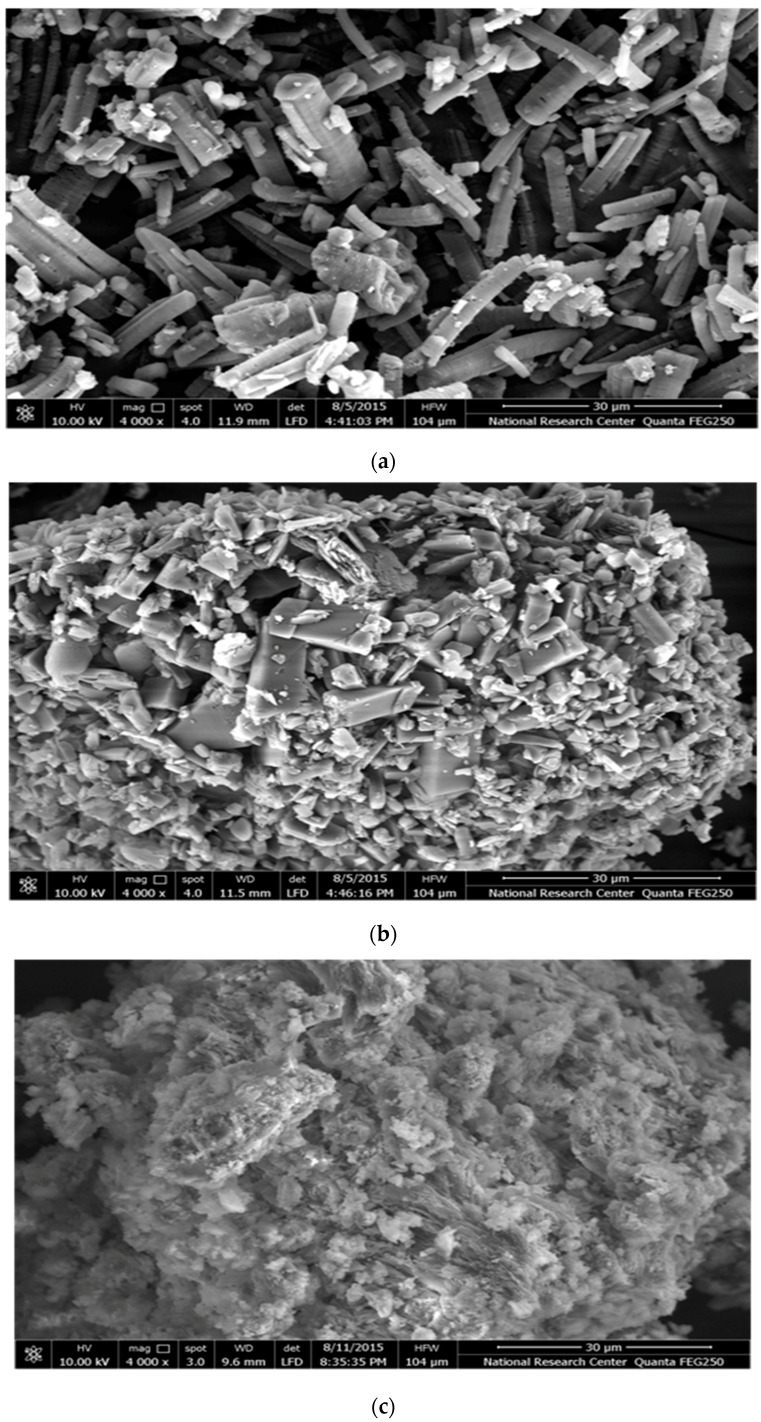

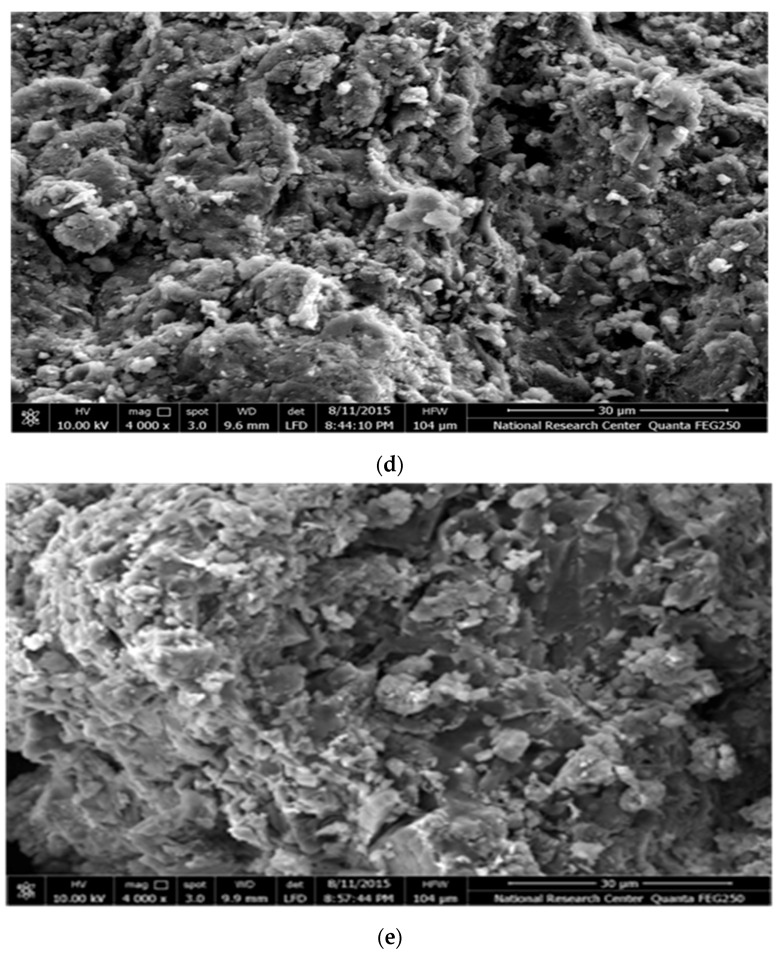
Scanning electron microscope of: fenoprofen calcium dihydrate (**a**); TA-β-CD (**b**); drug-TA-β-CD complex 1:3 (**c**); HPMC/drug complex (**d**); EC/drug complex (**e**).

The shape and surface morphology of fenoprofen calcium dihydrate, drug complex, HPMC/drug complex and /drug complex are shown in Figure 6. Fenoprofen calcium dihydrate and TA-β-CD have fairly different morphological characteristics. Fenoprofen calcium dihydrate appeared as tetrahydral crystals with smooth surfaces and homogeneous size. The TA-β-CD SEM examination revealed the existence of rhomboidal shape crystals with different dimensions [18].

The existence of unmodified particles of TA-β-CD in the drug complex was shown. That was automatically covered by a few crystals of drug. Detection of the features of the crystals of both components was hard, as only one type of crystal had appeared. The partial solubilization of TA-β-CD is the main explanation of such behavior, which modifies the adhesion property of FCD crystals to its surface.

A notable change in the morphology of the materials was revealed in the HPMC/drug and EC/drug complex systems. These systems were characterized by the existence of particles, partly broken, typical spherical shape and a smooth coating layer with some fissures. An observation of a creation of spherical particles aggregations was recorded. Actually, the physical appearance, morphology and size of the HPMC/drug complex and EC/drug complex products were totally unlike from those of the FCD, and it was not possible to distinguish the distinctive crystals of FCD, TA-β-CD, HPMC and EC. These observations, even though hardly conclusive, guide us to predict the presence of a single phase in the HPMC/drug complex and EC/drug complex systems [59].

### 3.3. In Vivo Studies

#### 3.3.1 Assessment of the Anti-inflammatory Activity

**Figure 7:**
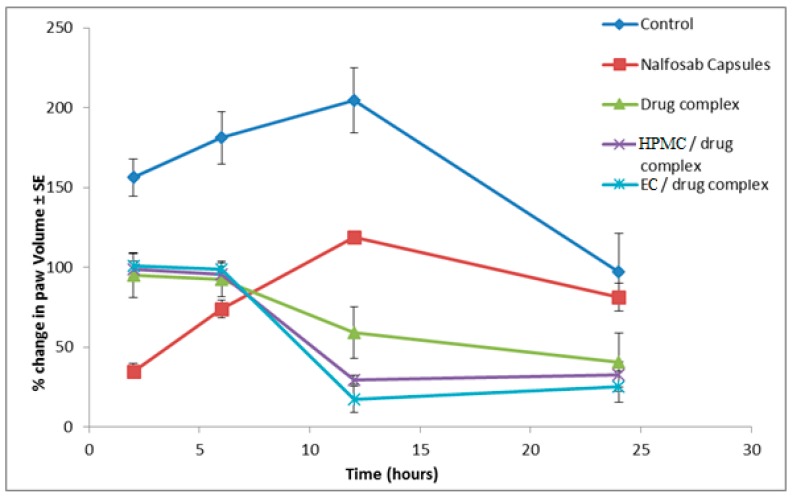
Effect of Nalfosab Capsules, drug complex, HPMC/drug complex and EC/drug complex on the carrageenan-induced acute edema in rat paw.

**Figure 8:**
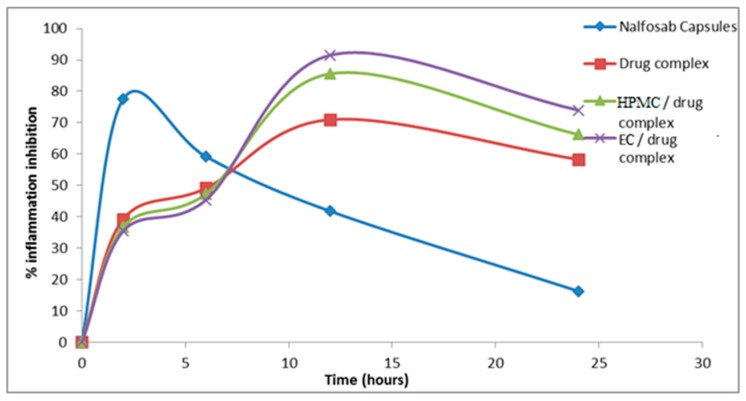
Mean % inhibition of induced inflammation by Nalfosab Capsules, drug complex, HPMC/drug complex and EC /drug complex.

The mean percentage change in rat paw volume (% edema) and the percentage inhibition of inflammation after administration of the three formulae and the marketed drug product (Nalfosab^®^ Capsules) were calculated and the data are presented in Figure 7 and Figure 8.

It is evident that the percentage of inhibition of inflammation−time profiles of the three formulae are quite different [62]. Two hours after the administration of the market product (Nalfosab^®^ Capsules), the % inhibition of carrageenan-induced rat paw edema was found to be 77.6% while it was 39.3%, 36.8% and 35.5% for drug complex, HPMC/drug complex and EC/drug complex, respectively. In the same pattern, there was a decrease in the % of inhibition of carrageenan-induced rat paw edema observed after six hours of Nalfosab^®^ Capsules post administration, while the other formulae (drug complex, HPMC/drug complex and EC/drug complex) showed an increase in the percentage of inhibition after six hours post administration. While in case of Nalfosab^®^ Capsules, after a 2 h time interval showing the maximum percentage of inhibition, then a decline in the percentage inhibition of edema was obtained at the 6, 12 and 24 h time intervals. In contrast, EC/drug complex showed higher percentage inhibition of the rat paw edema induced by carrageenan after both 12 and 24 h. This can be inferred to be due to prolonged release formulation/effect of EC/drug complex and in turn indicates its better anti-inflammatory effect 24 h post-administration as compared with Nalfosab^®^ Capsules and even drug complex or HPMC/drug complex. A significant difference (*p* < 0.05) between the percentage inhibition of inflammation of drug complex and Nalfosab^®^ Capsules was observed at all time intervals. In case of EC/drug complex and HPMC/drug complex, the significant difference as compared with Nalfosab^®^ Capsules was also obtained at all time intervals. There was a significant difference between the percentage change in rat paw volume of both the market product and the other formulae in comparison with the control group during all chosen time intervals.

Concerning the area under the percentage inhibition of inflammation–time curve (AUC_0-24_), calculated using the trapezoidal rule as a measurement of therapeutic effectiveness, EC/drug complex possesses the highest therapeutic efficiency as compared to Nalfosab Capsules followed by HPMC/drug complex and finally drug complex [63]

The *T*_max_ value of the drug complex, HPMC/dug complex and EC/drug complex showed higher value (12 h) compared to Nalfosab Capsules (2 h). This would indicate that these formulae exhibit a slower onset of action and the finding that points to their strongest anti-inflammatory effect being 12 h after administration as compared to Nalfosab Capsules. This finding states that both polymer/drug complexes express a sustained effect, which can be attributed to the sustained effect that was clearly demonstrated in vitro.

#### 3.3.2 Assessment of the Analgesic Activity

**Figure 9:**
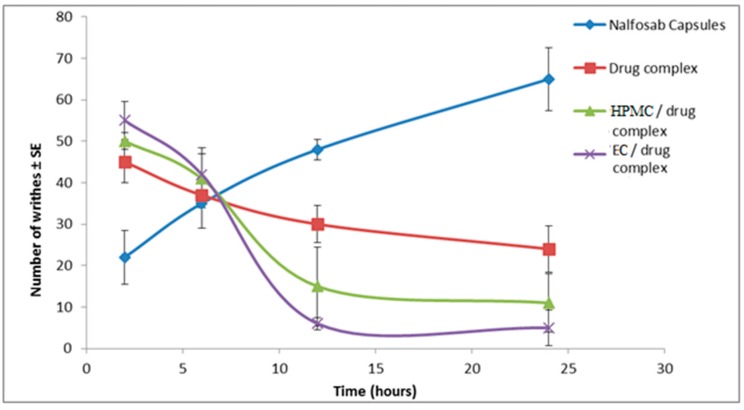
Effect of Nalfosab Capsules, drug complex, HPMC/drug complex and EC/drug complex on acetic acid induced abdominal writhing in mice.

**Figure 10:**
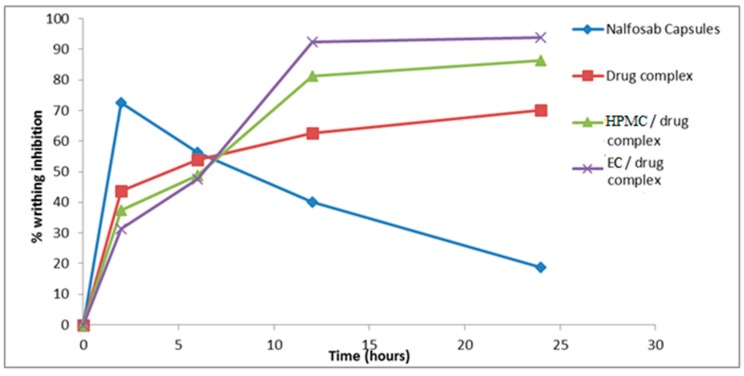
Mean % inhibition of induced abdominal writhing by Nalfosab Capsules, drug complex, HPMC/drug complex and EC/drug complex.

Results are shown in Figure 9 and Figure 10, which display the number of writhes observed and the percentage of inhibition reached by each of the examined formulae and the commercial Nalfosab^®^ Capsules.

It is obvious that Nalfosab^®^ Capsules and all of the three examined formulae significantly reduce the number of writhes compared to control (*p* ˂ 0.05) at all time intervals. There was a significant difference (*p* > 0.05) between the percentage inhibition of the market product and the other formulae at all time intervals. It is observed that EC/drug complex showed the maximum inhibition (92.5%, 93.8%) at the intervals of 12 and 24 h, followed by HPMC/drug complex (81.3%, 86.3%) then drug complex (62.5%, 70 %) and the least was the commercial product (40%, 18.8%). This in turn indicates that EC/drug complex has a sustained effect due to the highly amount of the drug remaining after the time had elapsed and therefore the highest percent of inhibition.

These results were in harmony with Agrawal et al. [64] who clearly demonstrated that the abdomen contraction stimulated by acetic acid that was inhibited by sumatriptan throughout the period of observation.

## 4. Conclusions

The lowest dissolution efficiency value was attained for the complex mixed with EC. DSC thermograms and IR spectra of drug/TA-β-CD complex, HPMC/drug and EC/drug complex indicate a possible interaction between the drug and TA-β-CD and between polymers and drug. X-ray patterns and SEM predict the presence of a single phase in the drug complex, HPMC/drug complex and EC/drug complex systems. EC/drug complex showed higher percentage inhibition of the rat paw edema induced by carrageenan after both 12 and 24 h as compared to the commercial product (Nalfosab^®^ Capsules) followed by HPMC/drug complex and finally drug complex. A remarkable inhibition of nociception demonstrated by EC/drug complex compared to Nalfosab^®^ Capsules was revealed, indicating that the EC/drug complex has a sustained effect due to slower release of drug over time due the polymer formulation.

## Abbreviations:

The following abbreviations are used in this manuscript:

**Abbreviation**	**Meaning**
%	Percentage
C	Degree Centigrade
µg	Microgram
µm	Micrometer
AUC	Area Under the Concentration Curve
CD	Cyclodextrin
C–H	Carbon Hydrogen bond
DE	Dissolution Efficiency
DSC	Differential Scanning Calorimetry
EC	Ethyl cellulose
FCD	Fenoprofen Calcium Dehydrate
FT-IR	Fourier Transform Infrared Spectroscopy
HPMC	Hydroxypropylmethyl Cellulose
Ip	Intraperitoneal
KBr	Potassium Bromide
KV	Kill Voltage
MC	Methylcellulose
M	Mole
MCE	Methocel Cellulose Ester
M_W_	Molecular Weight
Ma	Milliamper
Mwt/Mwt	Molecular Weight/Molecular Weight
N	Normal
Nm	Nanometer
OH	Hydroxyl
Ph	Hydrogen Ion Concentration
r.p.m	Rotation Per Minute
T_max_	Time Of Maximum Plasma Concentration.
TA-β-CD	Triacetyl-β-Cyclodextrin
USP	United States Pharmacopeia
V	Vibration
V–Me	Vibrational–Methyl
V–OH	Vibrational–OH
W	Watt
W/V	Wight/Volume
W/V	Wight/Volume
W/W	Weight/Weight
*λ*_max_	Maximum Wavelength
